# Comparative analysis of the postoperative effectiveness of different splenic artery aneurysm embolization techniques

**DOI:** 10.3389/fcvm.2026.1726290

**Published:** 2026-06-17

**Authors:** Biao Wu, Liang Chen, Xiaonan Wang, Hao Zhang, Jiang Zhu, Kangjie Chai, Liangxi Yuan

**Affiliations:** Department of Vascular Surgery, Changhai Hospital, Shanghai, China

**Keywords:** effectiveness, mushroom technique, post-embolization syndrome, sandwich technique, splenic artery aneurysms

## Abstract

**Purpose:**

The aim of this study was to compare the postoperative effectiveness of different embolization techniques for splenic artery aneurysms(SAAs), i.e., sandwich technique and mushroom technique, by analyzing data from our single center.

**Materials and methods:**

Between January 2019 and December 2023, a total of 220 patients with SAAs underwent embolization (sandwich technique *n* = 102, mushroom technique *n* = 118). Outcomes assessed included technical success, procedure time, length of hospital stay, total hospital costs, 30-day mortality, incidence of post-embolization syndrome(PES), splenic infarction rate, and aneurysm recanalization rate. Univariate and multivariate analyses were used to identify risk factors for PES in patients with splenic artery aneurysms after surgery. Normograms were used to predict the likelihood of developing PES after surgery.

**Results:**

The technical success rate of both embolization techniques was 100%, and the 30-day mortality, splenic infarction rate, and aneurysm recanalization rate were all 0. However, the mushroom technique was associated with less procedure time, length of hospital stay, total hospitalization costs, and incidence of PES than the sandwich technique. Univariate and multivariate analyses showed that the number of aneurysms as well as the embolization method were independent risk factors for PES.

**Conclusion:**

The mushroom technique achieves the same favorable short-term prognosis of the sandwich technique and results in a relative reduction in operative time, length of hospital stay, total hospital costs, and incidence of PES.

## Introduction

Splenic artery aneurysms (SAAs) are the most prevalent form of visceral artery aneurysms, constituting over 50% of all such aneurysms (1), with an incidence second only to abdominal aortic and iliac artery aneurysms (2). SAA is characterized by an artery dilation exceeding 1.5 times its normal diameter (3). Due to advancements in imaging technology and heightened public awareness of health check-ups, the detection rate of SAAs has markedly risen (4). Traditionally, open surgical procedures have been used for the treatment of SAAs; however, with the continuous improvement and maturation of endovascular techniques and devices (5, 6), endovascular approaches have progressively become the preferred treatment modality for SAAs (7, 8).

Endovascular techniques include embolization and reconstruction methods. Embolization has emerged as the preferred endovascular treatment for SAAs, owing to its straightforwardness and effectiveness. The sandwich technique, which utilizes coils to simultaneously occlude the inflow artery, the aneurysm sac, and the outflow artery concurrently, has garnered widespread acceptance among vascular surgeons for its effectiveness in occluding the aneurysm and its feeding arteries. However, some aneurysms, due to anatomical constraints, can only undergo embolization of the aneurysm sac and inflow artery. The postoperative effectiveness and effectiveness of mushroom technique remain unclear. The objective of this study is to undertake a retrospective analysis of 220 patients diagnosed with SAAs who underwent endovascular embolization at our center. The study will compare the effectiveness of the mushroom technique with the sandwich technique.

## Methods

### Patients

This retrospective study analyzed 220 patients with SAAs who received endovascular treatment at the First Affiliated Hospital of Naval Medical University between January 2019 and December 2023. All patients were diagnosed via computed tomography angiography (CTA) or digital subtraction angiography (DSA). Inclusion criteria: (1) aneurysms located in the main splenic artery with a diameter greater than 2 cm; (2) aneurysms located in secondary branches of the splenic artery with a diameter greater than 1 cm in symptomatic patients or women of childbearing age; (3) asymptomatic aneurysms with an annual growth of more than 0.5 cm; (4) asymptomatic patients with a desire for pregnancy; (5) symptomatic patients regardless of aneurysm size. Exclusion criteria: (1) coexisting diseases that could affect prognosis, such as significant organ dysfunction or tumors; (2) patients who underwent stent placement or stent-assisted coil embolization; (3) patients who underwent sac embolization or splenic artery embolization; (4) pseudoaneurysms; (5) dissecting aneurysms; (6) ruptured aneurysms; (7) missing data. This study was approved by the review board of the First Affiliated Hospital of Naval Medical University, and the informed consent was waived due to the retrospective nature of the study. The medical records were independently extracted and reviewed by two authors. The collected data included patient demographics, comorbidities, treatment strategies, and perioperative outcomes. Additionally, we evaluated aneurysm characteristics, such as size, shape, location, and quantity.

### Intervention

Preoperatively, all patients received relevant examinations, including CTA, Doppler ultrasound, or magnetic resonance imaging (MRI). Some patients with renal insufficiency underwent MRI. Experienced vascular surgeons comprehensively assessed patients' surgical history, groin infections, and vascular stenosis or occlusion to determine the appropriate surgical approach. The common femoral artery was the preferred access site; however, if the iliac artery exhibited severe tortuosity or if the angle between the celiac trunk and the aorta was small, the left brachial artery was considered as an alternative. All patients underwent modified Seldinger technique for puncture. A 0.035-inch guidewire was used to place a 5F pig-tail catheter (Cordis, Cardinal Health, Dublin, Ireland) into the abdominal aorta for angiography. The origin of the celiac trunk was identified, and a Cobra catheterr (Cook Medical, Bloomington, IN, USA) was navigated into the celiac trunk, followed by placement of a long sheath at the origin of the splenic artery. Angiography was performed to evaluate the course of the splenic artery, collateral vessels, and the aneurysm's position, morphology, and size. Interventions included: (i) coil embolization of the outflow artery, aneurysm sac, and inflow artery; (ii) coil embolization of the aneurysm sac and inflow artery.

### Definitions and follow-Up

Technical success was defined as the successful placement of coils during the procedure, and was angiographically defined as Raymond-Roy Grade I or II. Post-embolization syndrome (PES) was defined as the occurrence of fever, abdominal pain, distension, or elevated white blood cell counts in the post-operative period. Surgical time was defined as the duration from arterial puncture to sheath removal. Sandwich technique was defined as coil embolization of the outflow artery, aneurysm sac, and inflow artery, while the mushroom technique was defined as coil embolization of the aneurysm sac and inflow artery. A follow-up CTA was performed one year postoperatively in order to assess for aneurysm recanalization or splenic infarction. All patients were followed until December 31, 2024.

### Statistical analysis

Statistical analyses were performed using SPSS 26.0 (SPSS Inc., Chicago, IL, USA). Baseline characteristics were described as numbers and percentages for categorical variables, or means and standard deviations (SD) for continuous variables. Univariate logistic regression analysis was employed to assess the predictive factors for PES, followed by multivariate analysis for significant variables. A *p*-value of <0.05 was considered to be statistically significant.

## Results

### Inclusion criteria

**Figure 1:**
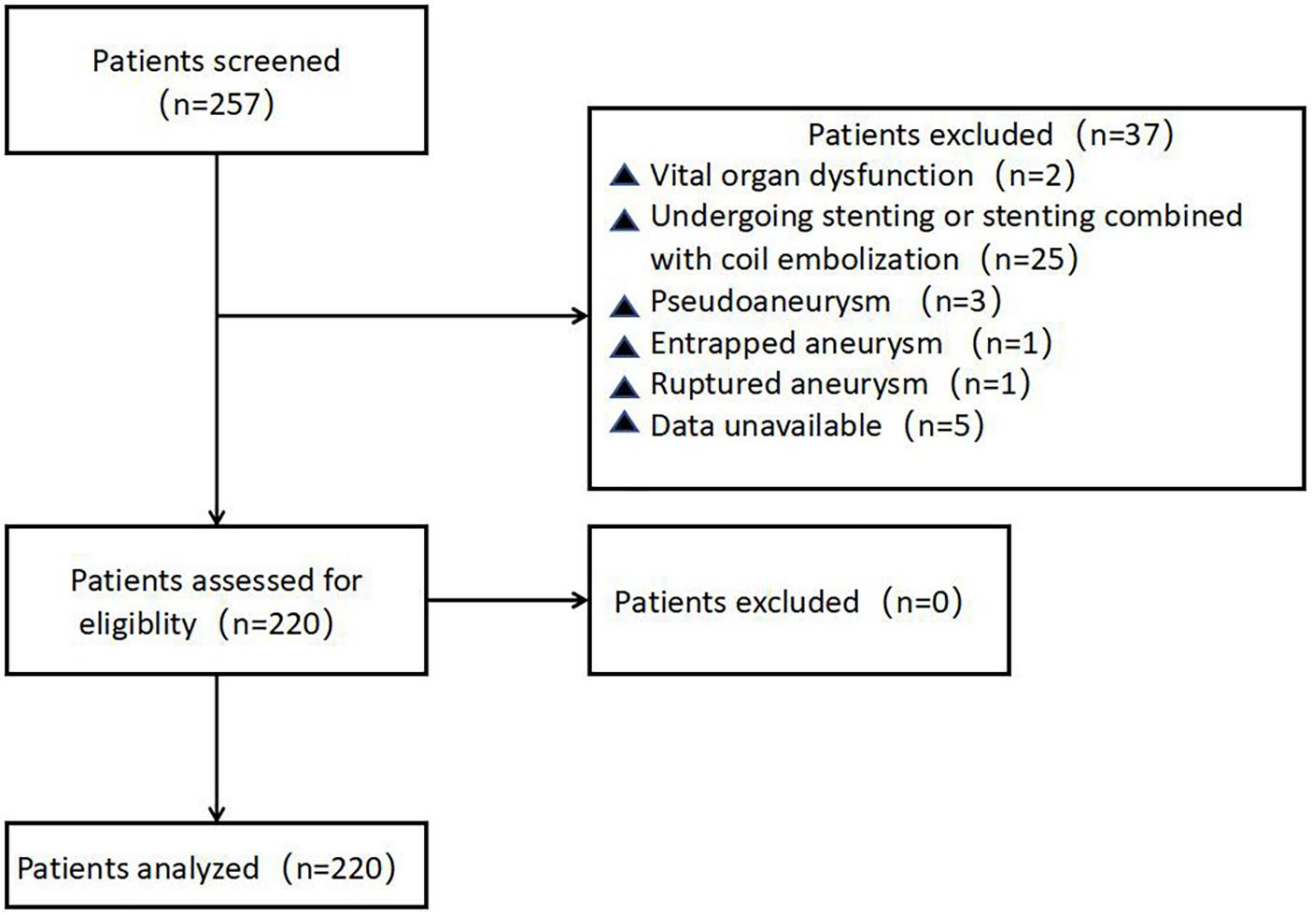
Patient selection process.

The flowchart for patient inclusion and exclusion is depicted in Figure 1. During the study period, a total of 257 patients with SAAs underwent endovascular surgery in our department, with 220 patients remaining after excluding those who did not meet the criteria.

### Baseline characteristics

The baseline characteristics of patients undergoing endovascular treatment for SAAs are detailed in Table 1. A total of 220 patients were included (150 females, 70 males; mean age: 58.2 years; range: 26–88 years). The average BMI was 24.1. 67 patients were smokers (30.5%), and 52 patients reported alcohol consumption (23.6%). Among them, 48 patients had symptomatic aneurysms. These symptoms include discomfort in the upper left abdomen, nausea or vomiting, and are diagnosed as symptomatic aneurysms only after excluding stomach, intestinal, and pancreatic diseases. Hypertension was the most common comorbidity (41.4%), followed by diabetes (15%). Other comorbidities included cerebrovascular disease (3.2%), coronary artery disease (3.2%), chronic kidney disease (1.4%), portal hypertension (4.5%), cirrhosis (4.5%), and other site aneurysms (8.2%). A history of previous abdominal surgery, such as appendectomy, cholecystectomy, or hysterectomy, was present in 62 patients.

**Table 1:** Baseline characteristics of patients.

Demographics	No.
Gender	
Male	70 (31.8%)
Female	150 (68.2%)
Age (years; mean ± SD)	58.2 ± 12.0
BMI	24.1 ± 3.64
Smoking	67 (30.5%)
Drinking	52 (23.6%)
Symptoms	
yes	48 (21.8%)
no	172 (78.2%)
Comorbidities	
Hypertension	91 (41.4%)
Diabetes	33 (15.0%)
Cerebrovascular Disease	7 (3.2%)
Coronary heart disease	7 (3.2%)
Chronic kidney disease	3 (1.4%)
Portal hypertension	10 (4.5%)
Cirrhosis	10 (4.5%)
Combination of other aneurysms	18 (8.2%)
History of abdominal surgery	62 (28.2%)

Data are presented as number (%) or mean ± standard deviation.

### Characteristics of splenic artery aneurysms

As shown in Table 2, there were 185 cases of single splenic artery aneurysms and 35 cases of multiple splenic artery aneurysms. The average diameter of the aneurysms was 23.82 mm. Aneurysm morphology primarily included saccular and fusiform shapes, with 114 and 106 cases respectively. 53 aneurysms exhibited calcification, and 11 had associated thrombus. Based on the location of the aneurysms in the splenic artery, they were categorized into proximal, middle, and distal/splenic hilum locations, corresponding to 39, 42, and 139 cases, respectively.

**Table 2:** Aneurysm characteristic and classification.

**Parameters**	**No.**
Single aneurysm	185 (84.1%)
Multiple aneurysms	35 (15.9%)
Mean diameter, mm	23.82 ± 10.57
Shape	
Fusiform	106 (48.2%)
Saccular	114 (51.8%)
Integrity	
Calcification	53 (24.1%)
Thrombosis	11 (5.0%)
Location of SAAs	
Proximal	39 (17.7%)
Middle	42 (19.1%)
Distal/spleen hilum	139 (63.2%)

Data are presented as number (%) or mean ± standard deviation.

### Perioperative and follow-up results

**Figure 2:**
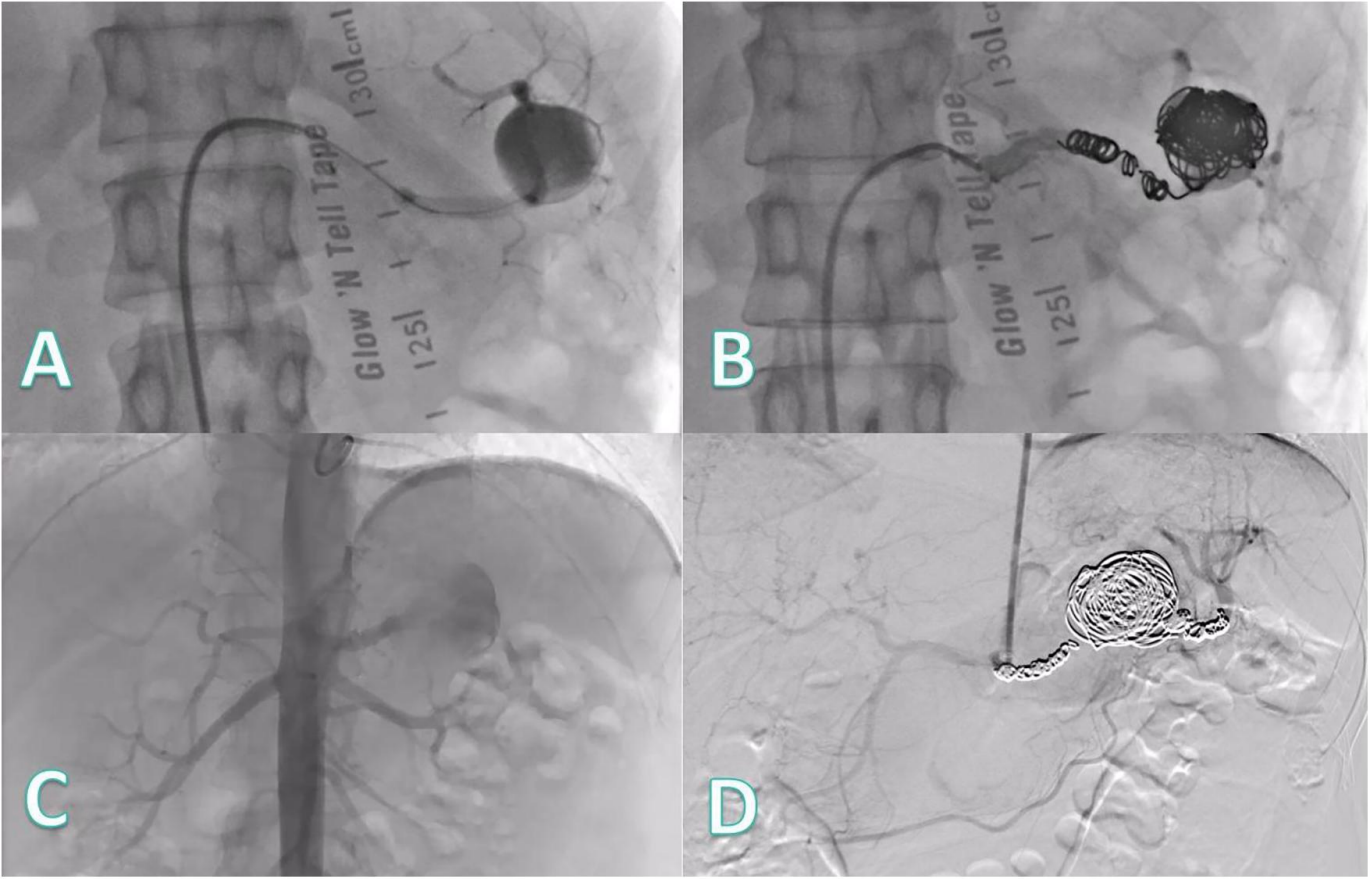
**(A,B)** mushroom technique, using coils to embolize inflow artery and aneurysm sac. **(C,D)** Sandwich technique, using coils to embolize outflow artery, aneurysm sac, and inflow artery.

Among the 220 patients, 118 and 102 patients utilised the mushroom technique (Figures 2A,B) and sandwich technique respectively (Figures 2C,D). As shown in Table 3, among the patients using the mushroom technique, 83 patients chose the femoral approach and 35 chose the brachial approach, with an average operative time of 30.5 min, an average hospital stay of 3.9 days, and an average total hospitalization cost of 43,370 RMB. Twelve patients developed PES after surgery. Among the patients using the sandwich technique, 76 patients chose the femoral approach and 26 patients chose the brachial approach, with an average operative time of 35.7 min, an average hospitalization time of 4.3 days, and an average total hospital cost of 58,851 RMB. Postoperative PES was seen in 38 patients. The success rate of both techniques was 100%. There were no deaths in the perioperative period or within 30 days of intervention. There was no aneurysm recanalization or splenic infarction in the 1-year postoperative follow-up CTA.

**Table 3:** Treatment and outcomes of 220 patients with SAAs.

Treatment and outcomes	Mushroom technique	Sandwich technique
Patients	118 (53.6%)	102 (46.4%)
Vascular access		
Transfemoral access	83 (70.3%)	76 (74.5%)
Transbrachial access	35 (29.7%)	26 (25.5%)
Technical success	118 (100%)	102 (100%)
Postembolization symptoms	12 (10.2%)	38 (37.3%)
Splenic infarction	0	0
Operation time–min	30.5 ± 10.3	35.7 ± 14.6
Hospital stay—d	3.9 ± 1.2	4.3 ± 1.6
Total hospital cost–¥	43,370 ± 20,445	58,851 ± 21,278
30-day mortality	0	0
Aneurysm revascularisation	0	0
Readmission rate	0	0
Follow-up length-year	3.1 ± 1.6	3.8 ± 2.1

Data are presented as number (%) or mean ± standard deviation.

### Correlation between surgical method and postoperative PES

In Table 4, univariate analysis revealed that patient age, the number of aneurysms, and the surgical method were associated with PES (*p* < 0.05), while other clinical and aneurysm characteristics showed no significant correlation (*p* > 0.05). Multivariate analysis indicated that the number of aneurysms and the surgical method were independent risk factors for the occurrence of PES in patients undergoing endovascular embolization for SAAs (*p* < 0.05).

**Table 4:** Univariate and multivariate analyses of characteristics with post-embolism syndrome.

Variable	Univariate		Multivariate	
Characteristics	HR (95% CI)	*P*	HR (95% CI)	*P*
Gender (male vs. female)	0.514 (0.228–1.161)	0.11		
Age (<50years vs. ≥50years)	2.836 (1.768–4.517)	**0**.**017**		
Aneurysm diameter (<30 mm vs. ≥30 mm)	0.554 (0.181–1.693)	0.3		
single or multiple aneurysms	5.312 (2.323–12.151)	**<0**.**001**	1.173 (0.075–1.683)	**<0**.**001**
Aneurysm location (proximal vs. middle vs. distal/spleen hilum)	1.179 (0.741–1.875)	0.488		
Aneurysm shape (fusiform vs. saccular)	0.626 (0.308–1.271)	0.195		
Surgical procedure (mushroom technique vs. sandwich technique)	3.903 (1.808–8.426)	**<0**.**001**	0.287 (0.076–0.532)	**0**.**014**

HR, hazard ratio.

Values in bold with *P*<0.05 are considered statistically significant.

## Discussion

SAAs are rare but potentially life-threatening conditions, with a female-to-male incidence ratio of 4:1 (9). The following risk factors have been identified: trauma, pregnancy, portal hypertension, atherosclerosis, advanced age, and female gender;Nevertheless, the pathophysiological mechanisms remain incompletely understood (10). Approximately 20% of patients present with symptoms, typically manifesting as left upper quadrant pain, often accompanied by anorexia, nausea, or vomiting (11). Aneurysm rupture is the most common and dangerous complication, leading to a mortality rate of 25% (12). Clinical manifestations include sudden abdominal pain and hemodynamic instability. Therefore, it is vital that early intervention is provided for patients with SAAs that meet surgical criteria. With the continuous development of endovascular techniques, which offer rare perioperative mortality and relatively low complication rates, endovascular treatment has gradually become the first-line approach for SAAs.

Currently, endovascular treatment options for SAAs primarily include stent placement, coil embolization, and stent-assisted coil embolization (2). Stent placement maximizes splenic artery blood flow and minimizes the incidence of splenic infarction (13). However, the conditions for stent placement are stringent; sufficient proximal and distal lengths of the aneurysm are required for good anchoring. Consequently, aneurysms located distally in the splenic artery or near the splenic hilum are unsuitable for stent placement. Additionally, an acute angle between the splenic artery and the celiac trunk may hinder the delivery of the stent system (14). Moreover, patients require long-term antiplatelet therapy following stent placement, which carries a risk of bleeding. Long-term complications may include stent migration or fracture, necessitating re-intervention (15).

Embolization is associated with common complications such as PES, characterized by fever, abdominal pain, and distension, which can be managed conservatively (16, 17). The most severe complication of embolization is splenic infarction or splenic abscess (18), which may require open splenectomy; however, the incidence of such complications remains low. This is attributed to the unique anatomical structure of the splenic artery, which possesses rich collateral circulation from branches such as the left gastric artery, left gastroepiploic artery, short gastric arteries, and small pancreatic branches, thereby reducing the risk of splenic infarction.

Currently, the most widely accepted embolization techniques include simple aneurysm sac embolization and the sandwich technique (19). Simple aneurysm sac embolization is limited to saccular aneurysms with narrow necks, thus presenting certain limitations. The sandwich technique is applicable to the vast majority of SAAs and is virtually contraindication-free, making it the most recognized simple and safe endovascular treatment (20).

The rationale for embolizing the outflow artery is to prevent retrograde blood flow that could lead to aneurysm enlargement and rupture. It is worth considering whether the pressure from retrograde blood flow in the outflow artery is sufficient to cause further enlargement of the aneurysm.

To explore whether embolization of the outflow artery is necessary for SAAs, we retrospectively analyzed data from patients who underwent endovascular embolization for SAAs at our center from January 2019 to December 2023. The mushroom technique was utilised in more than half of the patients for various reasons, including the proximity of the aneurysm sac to the splenic hilum, short outflow arteries, and the necessity to protect collateral vessels of the splenic artery, making coil deployment unsuitable. Furthermore, larger aneurysm sacs posed challenges for guidewire advancement to establish catheter access to the outflow artery. The selection of technique was also influenced by variations in surgical philosophy among operators and economic factors. The findings of this study demonstrate that both surgical methods attained a technical success rate of 100%. In this study, detachable coils were used for dense embolization, which allows for better control and precise deployment, making them the most commonly utilized materials. By selecting appropriately sized coils, the risk of coil displacement is minimized compared to other embolic agents such as biological glue or tissue adhesive (21). Liquid embolic agents are more susceptible to displacement under hemodynamic forces, potentially leading to occlusion of distal splenic artery collateral vessels and increasing the risk of splenic infarction or abscess. The data indicates that no patients in either surgical group experienced splenic infarction. However, CTA follow-up occurred one year postoperatively, and shorter-term follow-up was not conducted. It is possible that some patients experienced asymptomatic splenic infarction that resolved during the year following surgery. Moreover, no patients exhibited aneurysm recanalization postoperatively, indicating favorable short-term outcomes for both surgical techniques. This may be attributed to the dense embolization achieved during the procedure. Yasumoto et al. reported that incomplete embolization of SAAs may lead to reperfusion and recurrence (22). In terms of short-term effectiveness, both techniques yielded satisfactory results.

Conversely, data from patients' hospitalization indicated that those undergoing the sandwich technique were more prone to experiencing PES. The development of PES is primarily attributed to vascular intimal injury, localized splenic ischemia, and foreign body reactions. Although these symptoms typically resolve in the short term, they may extend hospitalization duration. Furthermore, the need to embolize the outflow artery necessitates the use of additional coils, thereby increasing surgical duration and costs. Multivariate analysis revealed that multiple splenic artery aneurysms and the sandwich technique were independent risk factors for PES in patients undergoing embolization for SAAs. Limitations

However, our study has several limitations. Firstly, although this is the largest single-center study to date on SAAs, further multicenter and large-scale studies are needed for more compelling validation. Secondly, the follow-up duration of this study was relatively short, necessitating longer follow-up to confirm the conclusions presented. Thirdly, we did not perform further stratified analyses based on aneurysm location and characteristics. Lastly, the early-phase imaging (CT or ultrasound) was not routinely performed.

## Conclusion

While the sandwich technique is currently recognized as an effective treatment for SAAs, the mushroom technique also achieves comparable short-term outcomes. Furthermore, it may reduce the incidence of postoperative PES, surgical duration, length of hospital stay, and total hospitalization costs. Factors such as splenic ischemia and necrosis, immune responses, absorption of metabolic products, alterations in splenic function, and individual variations, as well as surgical techniques, should be considered in future studies.

## Data Availability

The original contributions presented in the study are included in the article/Supplementary Material, further inquiries can be directed to the corresponding author.
